# Palmar-plantar erythrodysesthesia associated with capecitabine chemotherapy: a case report

**DOI:** 10.11604/pamj.2015.21.228.7525

**Published:** 2015-07-30

**Authors:** Gabriel Kigen, Naftali Busakhala, Evangeline Njiru, Fredrick Chite, Patrick Loehrer

**Affiliations:** 1 Department of Pharmacology &Toxicology, Moi University School of Medicine, Eldoret, Kenya; 2 Department of Haematology and Oncology, Moi University School of Medicine, Eldoret, Kenya; 3 Department of Medicine, Moi University School of Medicine, Eldoret, Kenya; 4 Indiana University Simon Cancer Center, Barnhill Dr, Indianapolis, United States

**Keywords:** Palmar-plantar erythrodysesthesia, capecitabine, Kenya

## Abstract

We report a case of a 62 year-old patient who developed Palmar-plantar erythrodysesthesia upon receiving four cycles of capacitabine-based chemotherapy. She was on post surgical adjuvant treatment for invasive well differentiated adenocarcinoma of the colon. The clinical and therapeutic aspects of this chemotherapeutic adverse effect are discussed.

## Introduction

Capecitabine is a chemotherapeutic agent that is widely used in the treatment of colorectal cancer. It a pro-drug of 5- fluorouracil (5-FU), and gets activated in the tumour tissue through a three step reaction mediated by thymidine phosphorylase enzyme. This enzyme, together with that responsible for the breakdown of 5-FU, dihydropyrimidine dehydrogenase are thought to contribute to the development of Palmar-plantar erythrodysesthesia (PPE), a chemotherapy adverse effect that is frequently associated with capecitabine therapy [1, 2].

## Patient and observation

**Figure 1:**
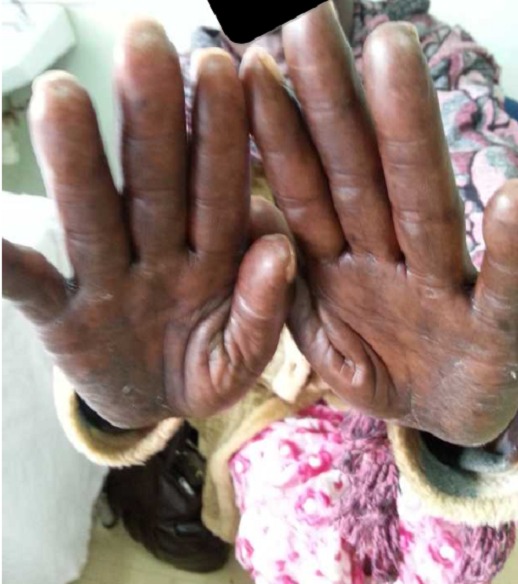
Blisters on the palms of the patient's hand

**Figure 2:**
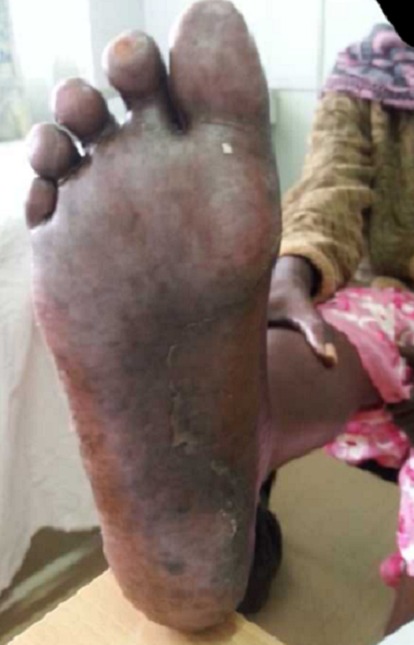
Blisters on the right foot

**Figure 3:**
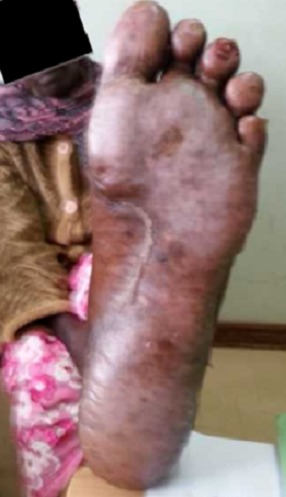
Blisters on the left foot

**Figure 4:**
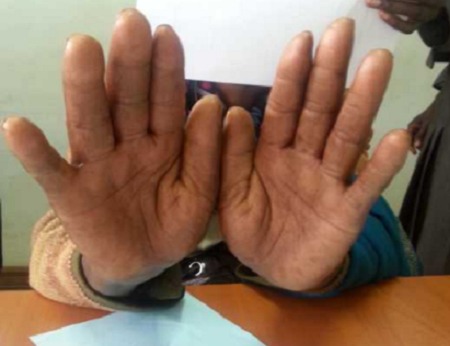
The patient's palms showing the healed blisters

**Figure 5:**
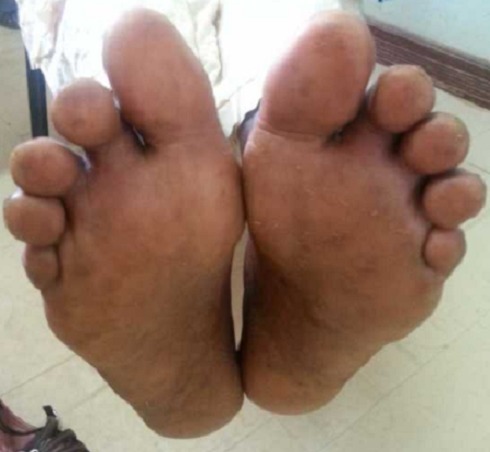
The patient's feet showing the healed blisters

A 62 year old female patient presented to oncology clinic with a diagnosis of colon cancer Duke C with liver metastasis, referred for adjuvant chemotherapy. She had initially been seen at her local hospital with a history of chronic constipation whereby hemicolectomy was done and a 19cm long colon was excised which included 3cm of tumour, 8cm from the distal resection margin extending to the serosal surfaces. Histology confirmed an invasive, well differentiated adenocarcinoma of the colon, Duke C with tumour extending into the serosa with lymphovascular and perineural involvement. Tumour deposits were also seen in the omentum. Resection margins and appendix were free of tumour, and no lymph nodes were identified. The Patient did well post operatively for two months before she presented to Moi Teaching and Referral Hospital with features of intestinal obstruction, which necessitated a repeat explorative laparatomy. Right hemicolectomy and omentectomy with lymph node dissection was done. Intra operatively, she was found to have liver metastatic lesions and omentum involvement. The patient was then referred to oncology clinic for palliative chemotherapy. At that point, the liver and renal function tests as well as complete blood count were within normal limits. The patient was then started on palliative adjuvant chemotherapy- XELOX regimen (oxaliplatin 120mgs and capecitabine 1500mgs twice daily), repeated three weekly. She received the first four cycles with no reported serious adverse events. She had only been on Vitamin supplements for the management of neuropathy after the third cycle. However, when she reported to the clinic for the 5^th^ cycle, she presented with symptoms of erythema, swellings, pain and blisters on the palms of the hands and soles of the feet (Figure 1, Figure 2, Figure 3). A diagnosis of PPE was arrived at, and the capecitabine-based chemotherapy discontinued. The patient continued on the Vitamin supplements and the lesions had healed after two weeks (Figure 4, Figure 5), and Carcino- embryonic antigen (CEA) test was normal (6.3ng/dl). The patient was then started on oxaliplatin- irinotecan therapy (IROX regime) after a two month-break from the time the syndrome resolved.

## Discussion

PPE, also referred to as hand-foot syndrome is a dermatologic adverse reaction that has been associated with some chemotherapeutic agents including docetaxel, cytarabine, doxorubicin, and fluorouracil and its pro-drug capecitabine [3]. It typically presents with dysthesias and tingling in hands and feet which may progress to blistering and ulceration [4]. The pathogenesis has not been well understood, but is thought to be due to damaged capillaries in the hands and feet leading to inflammatory reactions which have been postulated to be cyclooxygenase (COX)-2 mediated [5, 6]. Genetic variation in genes responsible for capecitabine metabolism have been reported to increase the risk of capecitabine-induced PPE [7, 8], and incidences of the adverse effect has been reported to be more common in black than white patients [9]. Treatment remains largely symptomatic, with treatment interruption and dose reduction being the most effective management currently [10–12].

## Conclusion

Early detection of the adverse effect, treatment interruption, patient education, conservative management and dose reduction or discontinuation of capecitabine therapy ameliorates the adverse effect in resource limited settings.
